# *IDH1* Targeting as a New Potential Option for Intrahepatic Cholangiocarcinoma Treatment—Current State and Future Perspectives

**DOI:** 10.3390/molecules25163754

**Published:** 2020-08-18

**Authors:** Fabiana Crispo, Michele Pietrafesa, Valentina Condelli, Francesca Maddalena, Giuseppina Bruno, Annamaria Piscazzi, Alessandro Sgambato, Franca Esposito, Matteo Landriscina

**Affiliations:** 1 Laboratory of Pre-Clinical and Translational Research, IRCCS, Referral Cancer Center of Basilicata, 85028 Rionero in Vulture (PZ), Italy; fabiana.crispo@crob.it (F.C.); michele.pietrafesa@crob.it (M.P.); valentina.condelli@crob.it (V.C.); francescamaddalena77@gmail.com (F.M.); alessandro.sgambato@crob.it (A.S.); 2 Medical Oncology Unit, Department of Medical and Surgical Sciences, University of Foggia, 71100 Foggia, Italy; giuseppina.bruno@unifg.it (G.B.); annamaria.piscazzi@unifg.it (A.P.); 3 Department of Molecular Medicine and Medical Biotechnology, University of Naples Federico II, 80131 Naples, Italy

**Keywords:** intrahepatic cholangiocarcinoma, isocitrate dehydrogenase, 2-hydroxyglutarate, *IDH1* inhibitors

## Abstract

Cholangiocarcinoma is a primary malignancy of the biliary tract characterized by late and unspecific symptoms, unfavorable prognosis, and few treatment options. The advent of next-generation sequencing has revealed potential targetable or actionable molecular alterations in biliary tumors. Among several identified genetic alterations, the *IDH1* mutation is arousing interest due to its role in epigenetic and metabolic remodeling. Indeed, some *IDH1* point mutations induce widespread epigenetic alterations by means of a gain-of-function of the enzyme, which becomes able to produce the oncometabolite 2-hydroxyglutarate, with inhibitory activity on α-ketoglutarate-dependent enzymes, such as DNA and histone demethylases. Thus, its accumulation produces changes in the expression of several key genes involved in cell differentiation and survival. At present, small-molecule inhibitors of *IDH1* mutated enzyme are under investigation in preclinical and clinical phases as promising innovative treatments for IDH1-mutated intrahepatic cholangiocarcinomas. This review examines the molecular rationale and the results of preclinical and early-phase studies on novel pharmacological agents targeting mutant *IDH1* in cholangiocarcinoma patients. Contextually, it will offer a starting point for discussion on combined therapies with metabolic and epigenetic drugs, to provide molecular support to target the interplay between metabolism and epigenetics, two hallmarks of cancer onset and progression.

## 1. Cholangiocarcinoma: From Classification to Treatment Strategies

Cholangiocarcinoma (CCA) is a heterogeneous group of hepatobiliary malignancy that originates from biliary epithelium, at any portion of the tree, and shows features of cholangiocyte differentiation [1]. Cholangiocarcinoma represents almost 3% of all gastrointestinal tumors and the global CCA incidence rate shows geographic variation, probably as a result of differences in genetic characteristics and/or exposure to risk factors of the world’s populations [2]. Intriguingly, Eastern countries, particularly the northeast of Thailand, exhibit higher age-standardized incidence rates (ASIRs) than in the West (Europe, United States and Australia), where the incidence of this disease is <6 per 100,000 cases [2], so much so that CCA is considered a rare cancer. Generally, a slightly smaller incidence and mortality is observed in women compared to men (the male-to-female ratio is 1:1.2–1.5) [3,4,5].

**Figure 1:**
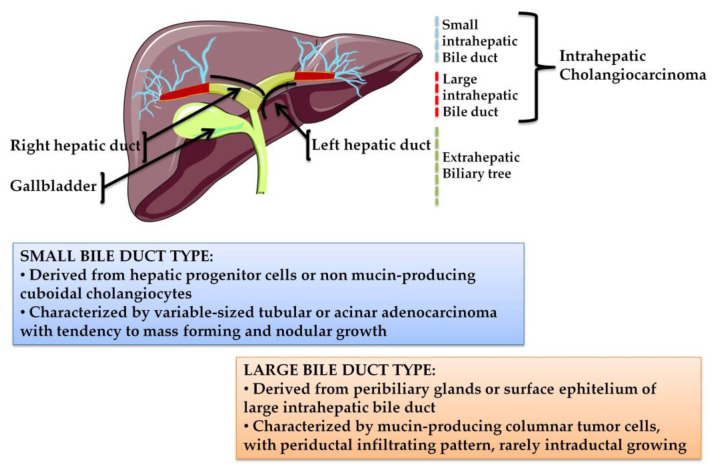
The biliary tree, based on anatomical classification, is subdivided into intrahepatic and extrahepatic portions. The extrahepatic tract comprises the right and left hepatic ducts, from which arises a perihilar CCA (pCCA), the common bile duct that accounts for distal CCA (dCCA), and the gallbladder. The intrahepatic counterpart derives from the second-order bile ducts. The sub-classification of intrahepatic CCA (iCCA) is based on the size of the duct from which the tumor originates: The small bile duct type arises from the interlobular and septal ducts, while the large duct type arises from the segmental ducts.

On the basis of their anatomical location, CCAs can be classified into three clinically distinct types of cancers: intrahepatic (iCCA), perihilar (pCCA), and distal (dCCA) cholangiocarcinoma (Figure 1).

iCCA arises in the second-degree bile ducts, specifically from segmental bile ducts to smaller branches of the intrahepatic part of biliary tree. In contrast, pCCA and dCCA are cancers of the extrahepatic biliary tree because the first originates from the right and/or left hepatic duct and/or the common hepatic duct proximally to the cystic duct origin, while the second occurs below the insertion of the cystic duct into the common bile duct, but not including the ampulla Vater [1,6]. pCCA accounts approximately 50–60% of CCAs cases and together with dCCA (20–30%) represents 80–90% of all CCAs diagnosed in the United States; the remaining 10–20% is represented by iCCA, which is the less frequent subtype of CCA, but the second most common primary intrahepatic malignancy after hepatocellular carcinoma (HCC) [5]. Although pCCA and dCCA represent the majority of CCA cases, over the last two decades, iCCA has shown a progressive increase of incidence in the world, whereas the incidence rates of other CCA subtypes have decreased in the same period [2,5].

This change in epidemiological trends is transforming the iCCA subtype into a global health problem that warrants attention and investigations for several reasons: (i) Incidence and mortality rates have risen significantly since the end of the past century; (ii) the knowledge about molecular mechanisms underlying iCCA onset is lacking and incomplete, thus a series of questions remain unanswered; (iii) this CCA subtype displays the highest inter-tumor heterogeneity, making its diagnosis complex, subsequently affecting the prognosis and management of patients; (iv) no effective therapies are available—thus far, iCCA is recognized as an orphan-drug disease.

Unlike pCCAs and dCCAs, which are mucin-producing adenocarcinomas (conventional type) or papillary tumors, iCCAs are characterized by highly variable morphological aspects, distinguishing a mucin-producing adenocarcinoma (bile duct (mucinous) type iCCAs or large bile duct type iCCAs), which originates from cholangiocytes and peribiliary glands, and a mixed subtype (bile ductular (mixed)-type iCCAs or small bile duct type iCCAs), in which areas of adenocarcinoma coexist with areas of hepatocytic differentiation, suggesting it originated from hepatic progenitor cells [2,6]. iCCA is characterized by clinical aggressiveness, like all CCA subtypes, but unlike pCCA and dCCA, it is adversely affected by several difficulties in patient management, probably due to its histological heterogeneity which results in divergent clinicopathological features. Less than 10% of patients survive over five year after diagnosis, mostly due to late diagnosis and limited treatment options [4]. Minimal and unspecific symptoms appear in early and/or pre-invasive stages, when surgical resection could represent the best therapeutic option combined with chemotherapy or radiation [3]. As a consequence, the diagnosis frequently occurs in the advanced stage of the disease, due to the “silent” clinical character of the tumor. Curative surgery with complete resection and/or liver transplantation is the preferred treatment option for iCCA, but only approximately 35% of patients are eligible for these approaches [2,4] and, after surgery, over half of resected patients have recurrence of disease in few years (generally 1–2 years depending on CCA subtype) [7]. Capecitabine as adjuvant chemotherapy showed efficacy in iCCA patients after surgical resection, with overall survival (OS) increased to 51 months in the treatment arm compared with 36 months in the observation arm (BilCap study). The PRODIGE12 study demonstrated that the adjuvant chemotherapy with gemcitabine and oxaliplatin (GEMOX), initiated three months after resection of biliary tract cancer, is ineffective in improving recurrence-free survival of CCA patients compared to placebo [8]. Other valid options for patients with localized unresectable CCAs are locoregional therapies, based on the focalized delivery of chemotherapy and radiotherapy. Techniques such as transarterial chemoembolization (TACE) and transarterial radioembolization (TARE), hepatic arterial-based therapies (HAT), radiofrequency ablation and photodynamic therapy (PDT) can increase survival and improve local control in locally advanced-metastatic iCCAs [9], although recurrence rates remain high [3]. In the field of innovative potential cancer treatment strategies, the use of non-toxic functionalized nanostructures, such as gold nanoparticles (AuNPs) [10] or graphene oxide nanosheets (GOxNs) [11], represent promising alternative systems to deliver chemotherapeutic agents selectively to tumor cells, reducing the drug exposure of normal cells. The potential application of these nanosized materials in targeted drug delivery and controlled release applications could improve the outcome of aggressive and poorly responsive cancers like iCCA.

For inoperable patients with metastasis disease at diagnosis, the median OS ranges from 5 to 12 months [3] and systemic therapies are the only curative opportunity, even though they have limited effectiveness [8]. The standard chemotherapeutic regimen for advanced iCCA is based on a gemcitabine-cisplatin combination (CisGem), which increases survival by about three months compared to gemcitabine alone, achieving an OS of 11.7 months [12]. However, this cancer is highly chemoresistant and, at present, no second-line therapy with established benefits is available, even though several studies were carried out for this purpose [13,14,15,16,17]. Chemoresistance is the major cause of treatment failure in this setting and is often caused by the activation of several anti-apoptotic pathways [18,19,20,21], which may act alone or synergistically to sustain cancer cell escape from cytotoxic or cytostatic effects of anticancer agents. Certainly, a better knowledge and/or comprehension of complex mechanisms responsible for drug resistance may improve the management of iCCA patients, suggesting targeted therapy strategies to enhance the response to chemotherapy [22].

iCCA is a lethal disease with an extremely poor response to conventional anticancer therapies and this consideration highlights the urgency of precision medicine approaches to go beyond current protocols. The advent of molecular profiling of human cancers and the discovery of driver mutations offers the opportunity to develop novel anticancer agents for clinical use, designed to hit specific molecular targets. In the iCCA subtype, few but distinctive genetic aberrations have been characterized; in particular, the isocitrate dehydrogenase 1 (*IDH1*) mutation, which is the most frequent, may represent the starting point for innovative individualized therapies. Indeed, several inhibitors of *IDH1* enzyme are available and they have already been tested in other malignancies with promising results [23]. In the scenario of personalized treatment, this review discusses the opportunity of using *IDH1* inhibitors as novel therapeutic options for *IDH1*-mutated iCCA patients and speculates on the advantage of their use in treatment as well as the potential of combined treatments for more successful approaches.

## 2. Molecular Profile of iCCA

The characterization of iCCA’s biological traits represents a milestone to better understand the complex framework of molecular alterations and improves the limits of the clinic-pathological classification. While some other human cancers benefit from targeted therapies that are widely accessible in clinical practice, at present no oncogene addiction has been defined for iCCA [24]. A plethora of papers has been published with the aim of identifying a genetic map, which could explain the mechanisms underlying iCCA’s pathogenesis, including rare and common genetic variants, chromosomal aberrations, and alterations of epigenomic and transcriptomic profiling [25]. Regarding genetic variants, the most prevalent in iCCA are *KRAS*, *BRAF*, *IDH1/2*, *EGFR*, *PTPN3*, *PIK3CA*, and loss of function *TP53* mutations. While *KRAS*, *TP53*, *EGFR*, and *PIK3CA* mutations are common in all CCAs, *BRAF*, *IDH1*/*2*, and *PTPN3* mutations prevail in iCCA [2]. Evidence of chromosomal aberrations, which results in both the loss and gain of gene copy numbers at different chromosomal loci, has been widely reviewed [24], but the best characterized is the *FGFR2* translocation and its related fusion proteins because of its potential amenability to targeted therapy [26]. The portion of the FGF receptor involved in translocation is the same in all CCA variants, determining constitutive activation of the downstream signaling pathway [27], but the fusion protein is almost exclusive of iCCA because these rearrangements are approximately absent in pCCA/dCCA and rare in HCC [27].

Investigating iCCA epigenomic profiling, several gene promoters have been found hypermethylated. Although it is not just a peculiarity of this cancer type, genes interested by epigenetic silencing are tumor suppressors involved in cell cycle control (e.g., *P16INK4A*, *P14ARF* and *RASSF1A*), regulation of cell adhesion/attachment and signaling (e.g., *CDH1*, *APC* and *SOCS3*), redox homeostasis (e.g., *GSTP*), and regulation of DNA damage (e.g., *MLH-1*) [28]. In addition, several chromatin remodeling factors, such as ARID1A, BAP1 and PBRM1, have been found with inactivating mutations [24], contributing to iCCA epigenetic rewiring.

Transcriptomic profiling allowed the sub-classification of iCCAs into two distinct molecular subclasses. The inflammatory subtype is enriched in IL-10/-6 and STAT3 pathway, while the “proliferation” subtype is characterized by the constitutive activation of RAS and MET signaling cascade [29]. Finally, emerging pathways are NOTCH signaling, which has a key role in the development of cholangiocyte [30], and WNT signaling, which achieves a central role in the development and progression of iCCA, as observed in other cancers [31]. Altogether, these alterations represent the current knowledge on both the genetic defects and pathways involved in the pathogenesis of iCCA and may provide putative candidates for targeted approaches.

### IDH1 Mutations in Intrahepatic Cholangiocarcinoma

A limited number of cancer types (e.g., glioma, acute myeloid leukemia and chondrosarcoma) harbor frequent mutations in *IDH1* and *IDH2* genes, among which iCCA is one. The expression of mutated forms of these genes is mostly absent in p/dCCA and HCC [32], thus *IDH1*/2 mutations could potentially be considered not only a therapeutic target for iCCA, but also a putative biomarker useful from a diagnostic perspective. *IDH1/2* mutations have been detected in 25% of iCCA cases. Even if some studies considered their combined frequencies [33], *IDH1* is more frequently mutated respect to *IDH2* and *IDH1* gene mutations, which range from 4.5 to 55.6% in iCCA [32], while the *IDH2* contribution is only partial, with a mutation rate that ranges from 2 to 6% [33]. Intriguingly, *IDH1* and *IDH2* mutations are mutually exclusive [32].

*IDH1* harbors missense mutations confined predominantly to a single residue (e.g., R132) in the active site of the enzyme. Five mutations have been described (i.e., p.R132H, p.R132C, p.R132G, p.R132S, and p.R132L) in *IDH1*-mutated cancers, but R132C is the most frequent in iCCA [32]. Among *IDH1/2*-mutated iCCA, the majority of cancers are poorly differentiated [34,35,36].

Considering the subset of *IDH1*-mutated iCCAs, a discrepancy was observed in the *IDH1* hotspot mutation frequency in two iCCA subtypes, small bile duct type and large bile duct type [6,37]. Hayashi et al. reported that only the small bile duct subtype harbors *IDH1/2* mutations and *FGFR2* translocation with an incidence of 21/53 and 6/53, respectively [38]. One small bile duct subtype case displayed co-occurring mutations in *KRAS* and *IDH1* genes [38]. Liau et al. obtained similar results observing a higher *IDH1/2* mutation rate (13/77) in cholangiolar-type, corresponding to the small bile duct iCCA, compared to the bile duct type, corresponding to the large bile duct iCCA [39]. In contrast, Akita et al. observed that the frequency of the *KRAS* mutation between the two groups of iCCA was not significantly different, but *IDH1* mutations remained specific for peripheral iCCA [40], and no mutation in *IDH2* was found in that cohort [40,41]. A similar stratification of iCCA subtypes based on mutational profiling was reported by Akita et al., who observed that *SMAD4* and *MDM2* mutations were restricted to pCCA and large duct type iCCA, whereas *BAP1* and *IDH1* were more specific to the small duct type iCCA [42], associated with lower CA19-9 levels [34,42]. Nevertheless, the clinical significance of lower level of CA19-9 in patients with IDH mutations needs to be clarified. No significant association of *IDH1/2* mutation with TNM stage, age or gender was reported in iCCA [34,35,36,43].

The mutation prevalence of *IDH1/2* in iCCA is mostly variable depending on the size and geographical location of the object of the studies [32]. The prognostic value of *IDH1* is still debated due to discordant results observed in different cohorts [32]. These discrepancies could depend on the distribution of risk factors in heterogeneous cohorts investigated in these studies. Indeed, the risk factors associated with iCCA, such as liver fluke infection and chronic viral hepatitis (e.g., HBV), are more prevalent in Asian countries than the West [44]. Jusakul et al. reported different genomic profiles among fluke-negative and positive iCCA, with the negatives characterized by *BAP1*, *IDH1/2* mutations, *FGFR* alterations and up-regulated PI3K signatures [45]. Chan-on et al. reported that *IDH1/2* genes were more commonly mutated in non-*Opisthorchis viverrini* flukes iCCA (Singapore cohort with 22.2% of cases) compared to *O. viverrini* iCCA (Thailand cohort with 3.2% of cases) [46]. Another study demonstrated a higher *IDH1/2* mutation prevalence in patients without hepatitis compared to those with hepatitis (20% vs. 2%, respectively) [47].

The advent of new technologies and high-throughput sequencing enabled genome mapping of iCCA patients, leading to the identification of a clear molecular iCCA subtype harboring *IDH1* mutations, with distinctive biological features. It also revealed a complex and highly variable genomic scenario in iCCA, which makes it difficult to define an unambiguous signature for this cancer type. Indeed, Lowery et al. described a tendency towards mutual exclusivity between genetic alterations such as *TP53*:*IDH1*, *IDH1*:*KRAS*, and *IDH1*:*FGFR2* [48], while the recent systematic literature review of Boscoe et al. showed that *IDH1* mutations were associated with other mutations of characteristic genes frequently found in iCCA [32]. Beyond *ARID1A* (22%), *BAP1* mutation or loss (13.3%); and *PBRM1* (13.3%), which were the most common, *PINK3A*, *CDKN2A*, *TP53*, *MAP2K1*, *SMAD4*, *KRAS*, *BRAF*, and *PTEN* showed a frequency ranging from 7% to 1% [32]. However, two independent studies found that *IDH1* and *BAP1* mutations were mutually exclusive [45,49].

Any conclusion about the differences observed in the frequency of mutations, association with prognosis and co-occurring/mutually exclusive mutation profiling must be taken with caution, due to cohort heterogeneity, a lack of rigorous inclusion criteria, and sampling difficulties due to the intrinsic and extrinsic heterogeneity of iCCA. Future studies are need for a better characterization of morpho-molecular subtypes of iCCA, which can be realized only if the identification of anatomical subtypes and clinicopathological features of the cohorts are scrupulously conducted. However, the discovery of *IDH1* mutations, even if restricted to an iCCA subtype, opens the way to use a precision therapeutic approach, potentially more effective than existent therapies, for the subclass of *IDH1*-harboring iCCAs.

## 3. Biological Impact of *IDH1* Mutation in Cellular Processes and Its Contribution to Carcinogenesis

**Figure 2:**
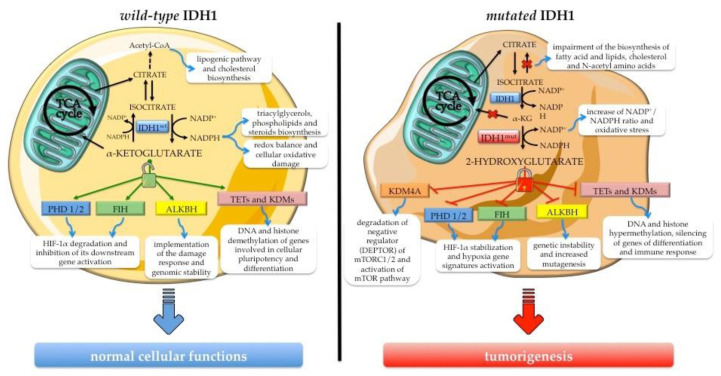
Overview of *IDH1*’s biological role in normal cellular functions and the consequence of its neomorphic activity acquired upon hot-spot mutation. The wild-type enzyme (*IDH1*^wt^) ensures normal cellular processes, sustaining metabolic pathways and the activity of α-ketoglutarate-dependent dioxygenases such as epigenetic enzymes (e.g., ten eleven translocation hydroxylases (TETs) and Jumonji C domain-containing lysine demethylases—JmjC-KDMs or JHDMs), HIF-1α regulators (e.g., factor inhibiting HIF (FIH) and prolyl hydroxylase domain-containing protein (PHD1/2)), and the DNA repair enzyme alkB homolog (ALKBH). The gain-of-function mutant enzyme (*IDH1*^mut^) produces the oncometabolite 2-hydroxyglutarate (2HG), a potent inhibitor of α-ketoglutarate-dependent dioxygenases, with concomitant depletion of the NADPH pool. The consequences of 2HG accumulation are the metabolic and epigenetic reprogramming and the aberrant activation of signaling pathways sustaining cancer onset and progression.

*IDH1* is a multifaceted enzyme with a crucial role in cellular metabolism, epigenetic regulation, cellular redox homeostasis, and DNA repair. Specific hot-spot mutations influence its catalytic activity, promoting a gain-of-function of the mutant form with aberrant and uncontrollable consequences on normal cell activities. Branching out into different physiological functions in the cells, *IDH1* mutations play a role in carcinogenesis processes, gaining value as novel targets for anticancer therapies (Figure 2).

*IDH1* is located in both the cytoplasm and peroxisomes, and catalyzes the reaction that leads to α-ketoglutarate (α-KG) production starting from oxidative decarboxylation of isocitrate (ICT). The reaction is reversible and dependent on nicotinamide adenine dinucleotide phosphate (NADP^+^), Mg^2+^ or Mn^2+^. *IDH1* acts as a homodimer and each monomer is characterized by three domains: The large domain (residues 1–103 and 286–414) characterized by a Rossmann fold; the small domain (residues 104–136 and 186–285) composed of an α/β sandwich structure; and the claps domain (residues 137–185) with two two-stranded and anti-parallel β-sheets [50]. *IDH1* has three biological conformational states, substantially differing in the structure of the active site. The regulation, from the transition active/closed conformation to the inactive/opened structure, is independent of post-transduction events; instead, a substrate-dependent self-regulatory mechanism controls this switch [50].

Regarding *IDH1* somatic mutations, no truncation or frameshift mutants have been reported and all mutations are heterozygous [51]. The mutations are confined to the catalytic site, especially at residue Arg132 (R132) level, leading to a gain of function of the enzyme [52]. This residue is responsible for forming hydrogen bonds with the α-carboxyl and β-carboxyl groups of isocitrate and mediates IDH-isocitrate binding [50]. The acquired mutation decreases the affinity for ICT with the concomitant increase of the binding affinity for NADPH. Thus, the *IDH1* mutant (*IDH1*^mut^) active site is shifted to reduce α-KG to d-2-hydroxyglutarate (d-2HG), acquiring a new catalytic function [52].

Although *IDH1* mutations with loss-of-function have been reported, all mutations harboring neomorphic activity produce the (D) isomer of 2HG [53,54]. Dang et al. proposed a model in which the wild-type/mutated *IDH1* (*IDH1*^wt^/*IDH1*^mut^) heterodimer catalyzes two reactions simultaneously: Conversion of ICT to α-KG reducing NADP^+^ by the wild-type monomer and conversion of α-KG to d-2HG with NADPH oxidation by the mutant monomer catalysis [52]. It has been demonstrated that wild-type *IDH1* (*IDH1*^wt^) gliomas are characterized by lower levels of d-2HG compared to heterozygous *IDH1*^mut^ tumors depending on the loss of the wild-type allele [55]. However, another study showed that the production of d-2HG is increased in *IDH1*^mut^ tumors if *IDH1*^wt^ is co-expressed [56]. Additionally, there is evidence that d-2HG concentration may vary among different IDH1 mutations [57,58]. R132G exhibits the highest levels followed by R132C and R132H [57,58], while R132S/L catalyze the conversion of α-KG to d-2HG at a rate similar to R132C/H [57].

2HG is an intermediary metabolite referred to as an oncometabolite for its oncogenic intracellular signaling functions [59] and because of its involvement in reprogramming of several cellular processes, such as gene expression and cellular metabolism. In nature, it exists as two enantiomers—*S*-2-HG (or l-2HG) and *R*-2-HG (or d-2HG)—generally produced at low concentrations in healthy mammalian cells through the activity of two FAD-dependent mitochondrial enzymes—d- and l-2-hydroxyglutarate dehydrogenase (D2HGDH and L2HGDH)—that convert the corresponding enantiomer into α-KG [60,61]. Several studies demonstrated that the aberrant accumulation of 2-HG within cells caused by neomorphic *IDH1*^mut^ activity or *IDH1*^wt^ transcriptional deregulation contributes to the onset of cancer [62].

### 3.1. The Role of Wild-Type IDH1 in Cellular Processes

The direction of *IDH1*-mediated reaction depends on physiological and/or environmental conditions and changes both the intracellular level of αKG and the NADP^+^/NADPH ratio, with remarkable consequences on a plethora of cellular pathways.

αKG is a metabolite with pleiotropic activity at the crossroad of a wide range of physiological activities beyond cellular metabolism [63]. It is an obligatory cofactor of numerous 2-oxoglutarate-dependent dioxygenases (2-OGDOs), a superfamily of phylogenetically conserved enzymes containing mononuclear non-heme iron sites that catalyzes hydroxylation and demethylation of proteins, which provide different biological functions [64]. In particular, some epigenetic effectors, such as the ten eleven translocation hydroxylases (TETs), enzymes promoting DNA demethylation, and the Jumonji C domain-containing lysine demethylases (JmjC-KDMs or JHDMs), acting on lysine residues of histone proteins, are members of 2-OGDO family and their activity is αKG-dependent. Thus, αKG availability influences and regulates epigenetic processes with consequences for the expression of some regulatory genes involved in cellular pluripotency and differentiation [65,66,67], as well as in oncogenic signaling correlated with stemness [68].

Other interesting members of 2-OGDOs are the prolyl hydroxylases (PHDs) and the factor inhibiting HIF (FIH, also known as HIF1AN), an asparaginyl hydroxylase. PHDs are involved in hypoxia inducible factors α-subunit paralog 1 (HIF-1α) destabilization by hydroxylation of conserved proline residues (Pro^402^ and Pro^564^), which triggers pVHL-mediated ubiquitination and the consequent proteasomal degradation of HIF-1α subunit [69]. Instead, hydroxylation of HIF-1α by FIH blocks its association with the transcriptional co-activators CREB-binding protein (CBP) and p300, preventing the activation of downstream genes [70,71]. Even modest variations of intracellular αKG abundance influence PHDs and FIH activity, leading to altered HIF-1α stabilization associated with profound changes in cells metabolism [72].

αKG is interwoven with DNA repair mechanisms due to the involvement of αKG-dependent dioxygenases in this cellular process. Indeed, the DNA repair enzyme alkB homolog (ALKBH) [73,74] and the DNA damage response proteins lysine-specific demethylase 4A/B (KDM4A/B) [75] require this metabolite to carry out their activity and implement damage response. Variability in cellular αKG pool, derived by glutamine catabolism, alters the steady-state DNA repair system with consequent genomic instability [76]. Other cellular processes for which αKG supply is important are fatty acid homeostasis [62] and the translational machinery [77], due to the involvement of some 2-OGDO enzymes [62,77].

Under hypoxia or mitochondrial metabolic impairment, *IDH1* activity becomes fundamental since cytosolic αKG is reductively carboxylate to generate citric acid and acetyl-CoA by the reversible *IDH1*-mediated reaction, to refill the lipogenesis pathway and the cholesterol biosynthesis of their precursors [78,79,80,81]. Beyond αKG, *IDH1* catalysis is the main source of non-mitochondrial NADPH, a crucial element of cell redox balance. This co-factor is a key reducing agent required for detoxification processes through the reduction of glutathione and thioredoxins, and the activation of catalase tetramer and cytochrome P450, which are together involved in cellular protection against reactive oxygen species [75]. In addition, as an electrons donor, NADPH is essential for the biosynthesis of triacylglycerols, phospholipids, steroids (e.g., cholesterol and steroid hormones), amino acids (glutamate and proline) and deoxyribonucleotides [82], in this way supporting the DNA damage repair system.

In this scenario, it is evident that *IDH1* acts at the intersection of different cellular processes and its dysregulation, in terms of both expression and activity, may be the starting point for the carcinogenetic process.

### 3.2. Biological Effects of Neomorphic Activity of Mutated IDH1

In the last decade, the attention on hot-spot mutations of *IDH1* has escalated because *IDH1*^mut^ produces d-2HG isomer that, despite being less potent than l-2HG in inhibiting some α-KG-dependent dioxygenases [83,84], has a notable impact on epigenetic reprogramming of *IDH1* mutated cells, due to the competitive block of TETs and JmjC-KDMs activity [83]. *IDH1*^mut^ cancers exhibit an hypermethylated phenotype characterized by CpG island hypermethylation and higher global DNA and histone methylation, in particular an increase in marks such as H3K4me3, H3K9me3 and H3K27me3 [85]. The biological effect of high DNA methylation and histone modifications of H3K9me3 and H3K27me3, associated with repression of transcription, is the silencing of genes involved in differentiation [86,87] and immune response [88,89]. Concerning the increase of H3K4me3, an activation mark, it could support the aberrant expression of the gene involved in oncogenesis and stem-like features [90,91,92], triggering carcinogenesis. Moreover, although genome methylation is generally considered a reversible and dynamic process, Turcan et al. demonstrated that the reprogramming of epigenome and transcriptome, driven by *IDH1*^mut^, shows partial persistence in specific genome regions even when the expression of mutated *IDH1* is suppressed [92]. This observation adds a new piece to the puzzle of *IDH1*^mut^ commitment to gene expression deregulation, which spreads to the control of chromatin domain formation. Indeed, beyond gene silencing, the increase of DNA methylation induced by 2-HG accumulation causes a rearrangement of chromatin domains and structures, such as insulator binding sites and/or enhancer-associated chromatin marks, leading to aberrant expression of oncogenes [93]. d-2HG may also predispose *IDH1*^mut^ cells to oncogenic transformation by direct inhibition of ALKBH 1 and 2, other α-KG-dependent dioxygenases involved in DNA repair pathways, and/or aberrant expression of DNA repair genes [94,95]. Although 2-HG produces genetic instability, which may contribute to cancer initiation by favoring mutagenesis, the accumulation of DNA damage could be an advantage for patients harboring *IDH1*^mut^ because it increases their vulnerability to chemotherapeutics and radiotherapy [75,85].

The mechanism underlying *IDH1* mutation and d-2HG tumorigenesis goes beyond DNA repair failure and epigenetic alterations. As a competitive inhibitor of 2-OGDO enzymes, d-2HG affects cellular signaling and pathways. Indeed, the oncometabolite inhibits two regulator enzymes of HIF-1 transcription factors, as mentioned above, even though conflicting observations have been reported. Some studies demonstrated a significant accumulation of HIF-1α [96] or β [97] in *IDH1*^mut^ cell lines, while others obtained contrasting results when examining the effect of 2-HG accumulation on HIF1 induction/stabilization and/or hypoxia gene signatures activation in different tumor types and in vivo models [94]. Additionally, Tarhonskaya et al. demonstrated a d-2HG-enabled activation of PHD2, assaying its activity in vitro. In a reducing environment and at physiologically relevant concentrations of Fe (II/III), the apparent activation of PHD2 by d-2HG is caused by a non-enzymatic conversion of 2-HG to α-KG [98]. It is likely that these HIF-1 results reflect different mechanisms for its stabilization depending on the cell type or contest/environment, and this may explain discrepancies in the literature. Moreover, it was demonstrated that d-2HG may contribute to enhanced HIF1 signaling directly by the inhibition of FIH [70] and/or indirectly by fault in collagen protein maturation [99]. Inhibition of collagen-4-prolyl hydroxylase by d-2HG reduces endostatin production [83], potentially favoring the induction of the hypoxia pathway. Indeed, endostatin, a secreted anti-angiogenic peptide generated by proteolytic processing of collagen XVIII within the extracellular matrix, downregulates HIF1 signaling reducing HIF1A expression and simultaneously increases FIH mRNA levels [100]. Additionally, in *IDH1*^mut^ cancers, collagen maturation defects were observed due to the 2HG-mediated hindrance to α-KG-dependent prolyl 4-hydroxylases 1, 2, and 3 (P4HA1/2/3), and procollagen-lysine 2-oxoglutarate 5-dioxygenases 1, 2, and 3 (PLOD1/2/3) activity [99]. This group of collagen hydroxylases mediates the hydroxylation of proline and lysine residues of collagen proteins driving their correct folding. An impairment of hydroxylysine-mediated glycosylation results in instability and major solubility of different type IV collagen proteins, with consequent fragility of vessels’ basal membranes, which could favor tumor progression by allowing epithelial cell invasion and angiogenesis [101].

Besides HIF1 signaling, another important pathway regulated by *IDH1*^mut^ is the mammalian/mechanistic target of rapamycin (mTOR), which is responsive to mitogenic signals and/or the availability of nutrients/cellular energy, and regulates cell growth, proliferation, autophagy, survival, and metabolism on the basis of signal inputs [102]. In various cell types with *IDH1* mutations, an unscheduled activation of mTOR pathway was found due to d-2-HG inhibitor effect on α-KG-dependent dioxygenases. Indeed, d-2HG-mediated inhibition of the lysine demethylase KDM4A causes destabilization of DEPTOR protein, the endogenous negative regulator of mTORC1/2, promoting its degradation by the proteasome and leading to mTOR being turned on in a PTEN-independent manner [103]. The physiological consequences of this unconventional activation of mTOR cascade are numerous and include cell growth and metabolism [104].

The acquisition of *IDH1* mutations results in substantial reprogramming of cellular metabolism. Reitman et al. demonstrated alterations in cellular concentration of several metabolites (e.g., some amino acids, glutathione metabolites, choline derivatives, and TCA cycle intermediates) for which the 2-HG production by *IDH1*^R132H^ neomorphic activity was not the unique factor responsible [105], but α-KG and NADPH availability became crucial in the rewiring of the metabolic landscape of cells carrying the mutation.

One of the most important pieces of evidence is that *IDH1*^mut^ is unable to catalyze the reductive carboxylation reaction and the glutaminolysis pathway becomes the pre-eminent source from which to generate α-KG for 2-HG in mutated cells [106]. Indeed, to compensate cytosolic α-KG depletion and increase d-2HG production, these cells redirect oxidative flux through the TCA cycle, increase respiration, and modulate the conversion of glutamine to citrate, acetyl-CoA, and fatty acids under various conditions, impairing the biosynthesis of fatty acid and lipids, cholesterol and *N*-acetyl amino acids [107]. In addition, the conversion reaction of α-KG to d-2HG consumes NADPH resulting in a change in the cellular NADP^+^/NADPH ratio that leads to an increase in oxidative stress by decreasing GSH pools [107]. The decrease of reducing equivalents may be compensated by transfers between the cytosolic and mitochondrial pyridine pools, so any alteration in redox homeostasis induced by low NADPH availability may also have implications for mitochondrial metabolism [106]. In addition, *IDH1* mutations may indirectly interfere with mitochondrial respiration, because d-2HG is able to inhibit the succinate dehydrogenase (SDH) enzyme. SDH inactivation causes an increase in cellular succinate levels and respiration inhibition with the concomitant onset of cancerous metabolism and mitochondrial dysfunction, leading to apoptosis resistance [108].

Ronen et al. reported the reprogramming of pyruvate metabolism in genetically engineered *IDH1*^mut^ cells via modulation of pyruvate dehydrogenase (PHD) [109], the enzyme that catalyzes the decarboxylation of pyruvate to acetyl CoA prior to entry into the TCA cycle. Via HIF1 signaling activation, *IDH1*^mut^ cells are able to reduce PHD activity, upregulating the expression of *PDK1* and *PDK3*, HIF1-regulated genes, which mediate the inhibitory phosphorylation of PHD. In this way, the flux of pyruvate into the TCA cycle is dropped; thus, the contribution of glucose to glutamate synthesis is reduced, as confirmed by the low levels of glutamate in mutant cells [105]. A decrease in glutamate is also obtained by 2HG-mediated inhibition of transaminases BCAT1 and BCAT2 in cells with *IDH1* mutations [110]. Cells could utilize excess glucose as an alternative source for 2-HG production [109]. As a matter of fact, glioma tumors and cells harboring *IDH1*^mut^ are characterized by lower intracellular lactate levels compared to *IDH1*^wt^ [111,112], probably due to hypermethylation of the lactate dehydrogenase A (*LDHA*) gene promoter [113] driven by 2-HG increase.

The tangled mechanisms by which *IDH1*^mut^ becomes able to interfere with normal cell functions remain unclear, but it is evident that the balance of intracellular levels of many factors, particularly d-2HG, αKG and NADPH, is crucial in contributing to cancer development and progression.

### 3.3. Biological Impact of IDH1 Mutation in Intrahepatic Cholangiocarcinoma

The biological defects of *IDH1* mutations have been investigated predominantly in gliomas and glioblastomas. Nonetheless, some downstream effects of both neomorphic enzyme activity and d-2HG accumulation may be seen in other cancer types with similar consequences on cellular physiopathology.

As previously reported, iCCA is one of the cancer types that frequently harbors mutations in the *IDH1* gene. The relatively small number of iCCA patients enrolled in studies and the difficulty for a correct discrimination between iCCA and other CCA subtypes has hindered the investigation of the biological role of *IDH1*^mut^ in iCCA carcinogenesis, and results are often contradictory in terms of prognostic significance. However, one indisputable observation is that iCCA patients with *IDH1*^mut^ are characterized by significantly higher levels of d-2HG, compared to wild-type tumors [114,115], and this may lead to alterations of normal cellular functions.

In 2013, Wang et al. demonstrated that *IDH1*^mut^/*IDH2*^mut^ iCCAs show a hypermethylated phenotype, at both the DNA and histone level, and half the hypermethylated genes found in iCCA overlap with epigenetically silenced genes identified in glioblastomas [116]. They speculated that some methylated genes could be involved in hepatocyte and cholangiocyte differentiation due to the higher levels of hepatic stem cell lineage markers in *IDH1*^mut^/*IDH2*^mut^ patients’ samples [116]. One year later, Saha et al. reported that an increase of 2-HG in hepatoblasts, expressing *IDH1*^R123C^ form, epigenetically controls the expression of HNF-4α, a master regulator of hepatocyte lineage progression [117]. They demonstrated the involvement of d-2HG in the inhibition of hepatocellular differentiation and uncontrolled proliferation of liver progenitor cells, with cooperative function of activated KRas, establishing that *IDH1*^mut^ may represent an early event in iCCA carcinogenesis, as observed in glioblastoma and acute myeloid leukemia (AML). During iCCA transcriptome reprogramming driven by *IDH1*/*2* mutations, an increase in levels of p53 protein was observed as a consequence of cellular stress induced by the elevated mutation rate; HIF-1α levels also increased, and this was caused by the 2-HG inhibitory effect on hypoxia regulator enzymes [116].

Recently, an additional role of *IDH1* mutations in CCA progression was established. Starting with a meta-analysis, Zhang et al. observed that the *P2RX7* gene is epigenetically regulated by *IDH1*^R132C^ mutation in CCA samples, demonstrating its involvement in cancer progression by affecting exosomes release from tumor cells [118].

Beyond DNA/histone methylome dysregulation, *IDH1*^mut^ is strictly associated with metabolic reprogramming in iCCA patients. Analyzing genomic (whole-exome sequencing, targeted exome sequencing) and epigenomic data from 496 iCCA patients, perturbation of purine, glutathione and citric acid pathways was revealed, suggesting an oncogenic function of *IDH1*^mut^, which implies cellular metabolism [119]. By an integrated analysis of CCA samples extracted by The Cancer Genome Atlas (TCGA), another research group identified a class of IDH mutant-enriched clusters with high expression of mitochondrial genes, including components of the electron transport chain and citric acid cycle, low expression of chromatin modifiers, in particular epigenetic silencing of ARID1A, and relatively high mitochondrial DNA copy numbers [120].

Additionally, an unexpected control of *IDH1*^mut^ on glycolytic flux has been observed in intrahepatic biliary organoids. The epigenetic remodeling prompted by oncogenic activity of the mutated enzyme causes an increase in H3K4me3 levels on the promoter region of *PFKP* gene with its consequent active transcription. The up-regulation of the rate-limiting glycolytic enzyme PFKP enhances glycolysis, promoting the formation of biliary organoids and conferring resistance to stress [121]. These results were confirmed by analyzing the expression of PFKP in surgically resected iCCA specimens: Elevated levels of enzyme were found in 68% of cases and the majority carried out *IDH1*^mut^ [121].

It is unclear whether hot-spot *IDH1* mutations are sufficient to support the initiation of the carcinogenetic process in iCCA or whether it needs to occur in combination with other genetic events, such as the activation of the Notch pathway and loss of p53 tumor suppressor [122]. Due to the rarity of these tumors, until now knowledge regarding the role of *IDH1*^mut^ in iCCA biology is limited and almost confined to epigenetic rewiring driven by 2-HG accumulation.

## 4. *IDH1* Inhibitors for Intrahepatic Cholangiocarcinoma Treatment

The discovery of gain-of-function mutations in the *IDH1* enzyme has revolutionized pharmaceutical approaches, making targeted therapies against mutated enzymatic forms a productive research field. Since mutations in *IDH1* occur in a broad spectrum of cancer types, both solid and hematologic, over recent years research has focused on the development of small synthetic molecules capable of inhibiting the aberrant activity of *IDH1*^mut^.

Many inhibitors are active against common *IDH1* mutations (e.g., R132H, R132C, R132G, R132S, and R132L), but few are specific for the *IDH1*^mut^ isoform (e.g., R140Q or R132C), and only one compound is a pan-inhibitor capable of blocking both *IDH1* and *IDH2* mutants. The majority of these small molecules acts as allosteric inhibitors, occupying the catalytic pocket of the enzyme in the open conformation, thus promoting structural changes that result in enzyme inactivity. However, other compounds bind specific amino acid residues, blocking the NADPH binding or chelating Mg^2+^/Mn^2+^ essentials for catalysis and for adopting the catalytically competent conformation [23].

Numerous pre-clinical and clinical investigations were conducted, particularly in AML and glioblastoma, and all of them demonstrated the positive effect of *IDH1*^mut^ inhibition on reversing epigenetic changes as a consequence of significant reductions of d-2HG levels. The biological effect of enzymatic inhibition included enhanced progenitor cell differentiation, decreased stem cell marker expression and a reduction of proliferation in both in vitro and derivative tumor models [62]. The successes obtained in clinical trials, in terms of both safety and efficacy, helped obtain FDA approval in 2018 of the first *IDH1* inhibitor—AG-120 (Ivosidenib), used to treat for refractory/relapsed AML adult patients harboring susceptible *IDH1* mutations [123].

Due to both the promising results obtained by *IDH1* inhibitors in clinical trials in other malignancies and the limitations in current treatment options for metastatic iCCA, *IDH1*^mut^ and its associated molecular pathways have become attractive therapeutic targets in the management of these patients. Four *IDH1*^mut^-inhibitors (e.g., pan-*IDH1*^mut^ AG-120 and BAY1436032; specific-*IDH1*^mut^ FT-2102 and IDH305) are under investigation for iCCA patients in six active clinical trials (Table 1). These molecules are chemically and pharmacodynamically different, although all of them are active against the same *IDH1* mutant isoform.

AG-120 (Tibsovo^®^, Agios Pharmaceuticals (Cambridge, MA, USA) is an oral allosteric reversible inhibitor active against various *IDH1*^R132^ mutants with comparable potency [124]. AG-120 is the result of a chemical structure optimization of the first *IDH1*-inhibitor AG-5198, a phenyl-glycine based compound with a strong inhibitory effect on the *IDH1* enzyme but poor metabolic stability. With the substitution of specific functional groups, AG-120 was obtained, a molecule characterized by high polarity and solubility; good stability in human liver microsomes; low human pregnane X receptor (PXR) activation, which regulates genes involved in drug metabolism and efflux; good permeability; and low efflux ratio [124]. At the dimer interface, the inhibitor competes with high specificity for binding with Mg^2+^ co-factor, a catalytically essential divalent ion, preventing the enzyme from achieving the catalytically competent conformation [129]. AG-120 also demonstrated slow-tight binding inhibition against the *IDH1*^wt^ homodimer [124] (Table 1), offering a new potential application in *IDH1*-overexpressing cancers.

At present, three clinical trials (NCT02073994, NCT02989857, NCT04088188) investigating AG-120 in CCA are underway. NCT02073994 is a Phase I multicenter open-label, dose escalation and expansion study, which enrolled patients with advanced solid tumors including CCA, 43.5% of the solid tumor cohort. The study has been conducted in order to assess the safety, tolerability, pharmacokinetics, pharmacodynamics and clinical activity of AG-120 as a single agent for treatment of patients harboring an *IDH1* mutation. Ivosidenib demonstrated rapid oral absorption and a long half-life, due to a slow elimination rate. After multiple doses at the optimum regimen of 500 mg administrated once daily (QD), patients with CCA showed plasma 2-HG levels decrease by up to 98%, a concentration similar to that seen in healthy subjects, and the inhibition effect was persistent over the whole treatment period [130]. In the CCA subgroup, AG-120 demonstrated good tolerability and no dose-limiting toxicities. Patients treated with ivosidenib at the regimen of 500 mg QD showed a median progression-free survival (PFS) of 3.8 months and a median OS of 13.8 months. Stable disease was noted in 56% of patients, which is clinically relevant considering that this result is comparable to the proportion of patients with stable disease receiving gemcitabine-cisplatin chemotherapy [131]. One limit of this study is represented by its non-randomized design, although Agios Pharmaceuticals sponsored a global Phase III randomized, double-blind, placebo-controlled study known as ClarIDHy (NCT02989857). This is intended to determine the efficacy and safety of orally administered Tibsovo^®^ (AG-120) in patients with previously treated *IDH1*-mutant CCA after progression on standard chemotherapy. The first results of this Phase III trial are encouraging because a significant increase of PFS for the ivosidenib group compared with the placebo group was observed. Under a regimen of 500 mg QD, patients treated with *IDH1* inhibitor showed a median PFS of 2.7 months compared to 1.4 months for the placebo group. Although this could appear a modest improvement in terms of absolute values, the results are statistically robust and indicate a notable reduction in risk of disease progression, independently of the number of previous lines of therapy. In addition, a difference of 4.8 months in median OS was observed between ivosidenib and the placebo group [132].

An independent non-randomized Phase I clinical trial was initiated in 2019 to assess the side effects and the optimal dose for treatment of unresectable and/or metastatic CCA patients harboring *IDH1* mutations, with gemcitabine and cisplatin in combination with AG-120 (NCT04088188). The study is still in its initial phase and is not yet recruiting.

IDH305 is a selective *IDH1*^R132H/C^ inhibitor (Table 1) developed by Novartis Pharmaceuticals (Basel, Switzerland). This pyrimidin-5-yl-oxazolidine-2-one acts as allosteric non-competitive inhibitor occupying the binding pocket of the enzyme and stabilizing *IDH1* mutant in a catalytically inactive open conformation via steric hindrance. In pre-clinical studies, this inhibitor showed an interesting brain penetration compared to other *IDH1*-inibiting compounds, low liver microsomal clearance values and comparable 2-HG reduction efficacy with respect to other inhibitors [125]. In 2015, a Phase I non-randomized clinical trial (NCT02381886) was started to estimate the safety and tolerability of IDH305, to assess the pharmacodynamics and plasma pharmacokinetic of the compound and evaluate the overall response rate in order to test the anti-tumor activity of this inhibitor. Results are not yet available but 166 patients with advanced malignancies harboring *IDH1*^R132^ mutations, including iCCA, have been enrolled.

Very few pre-clinical studies are available regarding FT-2102 (olutasidenib), a potent, orally active and brain penetrant inhibitor of *IDH1*^mut^ (Table 1), the development of which was supported by Forma Therapeutics (Watertown, MA, United States). FT-2102 is an optimized quinoline with potent and selective inhibitory activity against *IDH1* mutants and a good oral bioavailability. This molecule efficiently reduces 2-HG production in xenograft *IDH1*^R132H^ in vivo models, binding competitively with the isocitrate-binding pocket of each monomer of *IDH1*^mut^, near the dimer interface, and blocking the conformational changes fundamental for catalysis. Moreover, FT-2102 showed good cell permeability and microsomal stability as well as low efflux ratio in xenograft models [126]. At present, FT-2102 is undergoing clinical investigation for advanced solid tumors and gliomas (NCT03684811). The study was initiated to assess the safety, efficacy, pharmacokinetic and pharmacodynamic of FT-2102 as a single agent and in combination with other anti-cancer drugs, among them the DNA-demethylating agent azacytidine. As a consequence, the clinical trial is planned to be structured in two parts: iCCA patients will be enrolled for a Phase I/II trial for dose determination and clinical activity will take place in the single-agent arm and/or in the gemcitabine/cisplatin combination arm.

BAY-1436032 is a potent pan-inhibitor equally active against all mutant isoforms of *IDH1* (Table 1), the development of which has been sponsored by Bayer AG (Leverkusen, Germany). Development was conducted upon screening of over 3 million of compounds. It performs its inhibitory effect in an allosteric manner, binding the pocket located between the two *IDH1*^mut^ molecules of the homodimeric *IDH1*^R132^ complex, and stabilizing the inactive open conformation [127]. The reduced 2-HG production induced by BAY 1,436,032 has a positive effect on proliferation and differentiation in primary glioma cultures [127] and in patient-derived AML mouse xenografts harboring *IDH1* mutations [128]. In 2016, an open-label, non-randomized, multicenter Phase I study (NCT02746081) began to evaluate the tolerability and safety of BAY1436032, and to investigate the pharmacokinetics and preliminary pharmacodynamic of this inhibitor in patients with *IDH1*^R132^-mutant advanced solid tumors, including *IDH1*-mutated iCCAs. The cohort of enrolled patients is still limited (88 patients); thus, preliminary results are lacking.

Two independent clinical trials are also being conducted in the European Union and have been registered in the EU Clinical Trials Register. One is a Phase 1b/2 study (EudraCT Number: 2018-001796-21) designed for the assessment of FT-2102 in a heterogeneous group of patients with advanced solid tumors, including iCCA, characterized by *IDH1* mutations. The second is focused on CCA; indeed, it is a Phase 3, multicenter, randomized trial (EudraCT Number: 2015-005117-72) activated to investigate the efficacy of AG-120 in the treatment of non-resectable or metastatic CCAs harboring *IDH1*^mut^.

## 5. Future Perspectives of *IDH1* Inhibitors Use in Intrahepatic Cholangiocarcinoma

The status of “rare cancer” and the difficulties for clinicians to obtain a correct molecular diagnosis for iCCA patients have contributed to the lack of adequate treatments for this type of aggressive tumor. In addition, conventional therapies (e.g., chemotherapy and radiotherapy) produced unsatisfying results, particularly in the adjuvant and second-line settings, supporting an immediate demand of effective alternative treatment options.

The advent of NGS technologies suggested that iCCA represents a subgroup of CCAs with discrete driver mutations, some of which are targetable with novel therapies. In particular, *IDH1* mutations have aroused increasing interest in the field of targeted therapies because of their involvement in both metabolic and epigenetic rewiring, two hallmarks of cancer interwoven with tumor initiation and progression [133]. Although the prognostic significance of *IDH1*^mut^ in iCCA is still debated [32,34], the promising results obtained by clinical trials are indisputable [132]. However, this field of investigation is still in its early phase and several issues are still debated. An unsolved open question is the potential contribute of *IDH1*^mut^ in favoring sensitivity to agents targeting specific pathways involved in cancer growth and progression. Indeed, in vitro studies revealed that iCCA cell lines harboring different hot-spot mutations *IDH1*^R132^ are more sensitive to dasatanib [134], a multi-tyrosine kinase inhibitor, JQ1 [135], a selective inhibitor of bromodomain and extraterminal domain (BET) proteins, and olaparib [136], a poly(adenosine 5′-diphosphate) ribose polymerase (PARP) inhibitor, when compared to wild-type lines. However, none of these studies looked deeply into the characterization of molecular mechanisms underlying the anti-tumor effects of these drugs on *IDH1*^mut^ iCCA cells, limiting their translational application. In fact, the phase II trial (NCT02428855), activated in 2015 to investigate the safety and effectiveness of dasatanib in CCA treatment, was closed because it was not approved by the FDA for this specific purpose.

Despite significant preclinical and clinical successes obtained by *IDH1*^mut^ inhibitors as single agents for the management of cancers with *IDH1* mutations, there is an opportunity to improve their efficacy. It was demonstrated that the global DNA hypermethylated pattern, driven by *IDH1* neomorphic activity, may not be rapidly and completely reversed upon single-agent inhibition of the mutated enzyme and/or depletion of 2-HG accumulation [92,137]. Consequently, it may be worthwhile to investigate combination therapies with *IDH1* inhibitors and demethylating and/or histone modifying agents in order to improve the beneficial effect of an *IDH1*^mut^ targeted monotherapy. In such a context, the NCT03684811 trial has already included a cohort of FT-2102 in combination with azacytidine but enrolling only glioma and chondrosarcoma patients. However, the encouraging utility of combining epigenetic drugs with small-molecule inhibitors of mutant *IDH1* emerged by several trials active for AML and glioblastoma, and this may provide a robust rationale to extend this strategy in advanced and metastatic iCCA.

A fascinating unexplored research field is the potential oncogenic role that *IDH1*^wt^ overexpression may play in iCCA pathogenesis. Indeed, a recent study by Su et al. highlighted that *IDH1*^wt^ (not the mutated form *IDH1*^R132C^) promotes cell proliferation, invasion and migration in iCCA cell lines by regulation of αKG and NADPH levels [138]. These results are not surprising because over recent years it has emerged that an aberrant expression of non-mutated *IDH1* in numerous cancers (e.g., non-small cell lung carcinoma, squamous cell lung cancer, pancreatic ductal adenocarcinoma, primary glioblastoma) [139,140,141,142] correlates with therapy resistance, an aggressive phenotype and poor prognosis [141,143,144,145]. The biological consequences of *IDH1* up-regulation are ascribable to the central role of this enzyme in the regulation of both metabolic and epigenetic processes. Recently, the *IDH1*^wt^ enzyme has become an interesting actionable target for cancer therapy equal to the mutated isoforms. Some developed *IDH1*^mut^ inhibitors, such as AG-120 and GSK321, are active against the wild-type enzyme at relatively low concentrations (*IDH1*^wt^ IC_50_: 0.046 μM vs. *IDH1*^mut^ IC_50_: 0.0046 μM for GSK321) [62], allowing their use for *IDH1*-overexpressing cancers as single agents or in combination with other anticancer therapies. To our knowledge, at present, investigations focused on *IDH1*^wt^ expression in iCCA have been not yet started, even though *IDH1*^wt^-dependent regulation of the expression of EMT markers was reported in CCA [138].

In conclusion, it is evident that *IDH1* enzyme is entangled involved in many cellular functions and is an interesting therapeutic option for orphan-drug cancer types such as iCCA. However, patients should be evaluated not only for the mutational profile of *IDH1* enzyme, but also for its expression and activity, in order to improve the applicability and effectiveness of *IDH1* inhibitors in advanced iCCA management.

## Figures and Tables

**Table 1:** *IDH1* inhibitors under clinical investigation in metastatic iCCA.

IC_50_ (µM)						
Drug	*IDH1* Mutation	Mutant	Wild-Type	Ref	Clinical Trial Number	Phase
AG-120	R132H R132C R132G R132L R132S	0.012 0.013 0.008 0.013 0.012	0.072	[124]	NCT02073994 NCT02989857 NCT04088188 2015-005117-72	I III I III
IDH305	R132H R132C	0.027 0.028	6.14	[125]	NCT02381886	I
FT-2102	R132H R132C	0.0212 0.0094	>20.0	[126]	NCT03684811 2018-001796-21	I/II Ib/II
BAY1436032	R132H R132C R132G R132L R132S	0.015 0.015 0.004 * 0.003 * 0.016 *	20.0	[127,128]	NCT02746081	

* IC_50_ evaluated on primary AML patient-derived cells [128].
